# Supplementary material for: In situ analysis reveals the TRiC duty cycle and PDCD5 as an open-state cofactor
Source: Nature. 2024 Dec 11;637(8047):983–90. doi: 10.1038/s41586-024-08321-z (PMC11754096; doi:10.1038/s41586-024-08321-z)
Supplement: Supplementary file 1 — Supplementary Figs. 1–10. [file 41586_2024_8321_MOESM1_ESM.pdf]

nature portfolio

https://doi.org/10.1038/s41586-024-08321-z

Supplementary information

In situ analysis reveals the TRiC duty cycle and PDCD5 as an open-state cofactor

In the format provided by the authors and unedited

Supplementary Information Guide

Supplementary Fig. 1: Data processing of TRiC in HHT-treated cells.

Supplementary Fig. 2: Validation of open and closed TRiC classification in two datasets.

Supplementary Fig. 3: Validation of classification of PFD-bound TRiC particles in untreated cells.

Supplementary Fig. 4: Classification of densities in the open TRiC chamber.

Supplementary Fig. 5: Classification of densities in the closed TRiC chamber.

Supplementary Fig. 6: The additional density from open TRiC fitted with TRiC binders.

Supplementary Fig. 7: Functional association of PDCD5 with the TRiC and its substrates.

Supplementary Fig. 8: Spatial analysis of TRiC and ribosome in human cells.

Supplementary Fig. 9: Validation of classification of PFD-bound TRiC particles in HHT-treated cells.

Supplementary Fig. 10: PDCD5 does not affect the ATPase activity of TRiC

Supplementary Video 1: The template matching of TRiC in a tomogram.

For visualization, the tomogram was set to 50% transparency, and the CCC value of TRiC was colored in yellow.

Supplementary Video 2: Three-dimensional views of the TRiC complex.

Cryo-ET maps of open and closed TRiC fitted with PDB 7X3J and 7NVN.

Supplementary Video 3: Three-dimensional views of CCT3-CCT1-CCT4-PDCD5.

The structure of PDCD5 (38-112 a.a.) in complex with the equatorial domain of CCT3-CCT1-CCT4 predicted by AlphaFold-Multimer was fitted into the segmented open TRiC map. PDCD5, cyan. CCT3, blue. CCT1, magenta. CCT4, yellow.

Supplementary Video 4: Different TRiC states were mapped back into a tomogram from the untreated cell.

Open TRiC without PFD, maroon. Open TRiC with 1 PFD, cyan. Open TRiC with 2 PFDs, brown. Closed TRiC, purple.

Supplementary Video 5: Examples of TRiC clusters in a tomogram.

Closed TRiC was colored in purple.

Supplementary Video 6: A tomogram shows TRiC and actin filaments.

Closed TRiC (purple), open TRiC (orange) and actin filaments (carnation) were mapped back into a tomogram from the untreated cell.

Supplementary Video 7: An example of TRiC close to the ribosome exit tunnel.

Closed TRiC, purple. Open TRiC, orange. Ribosome, teal.

Supplementary Video 8: 3D views of ribosomes and TRiC in a tomogram.

Closed TRiC (purple), open TRiC (orange), monosomes (teal), polysomes (strawberry), 60S (aqua) and 40S (yellow) were mapped back into a tomogram from the untreated cell.

Supplementary Video 9: Three-dimensional views of PFD.

PFD (PDB 7WU7) was fitted into the corresponding densities segmented from the TRiC map bound with one PFD in HHT-treated cells.

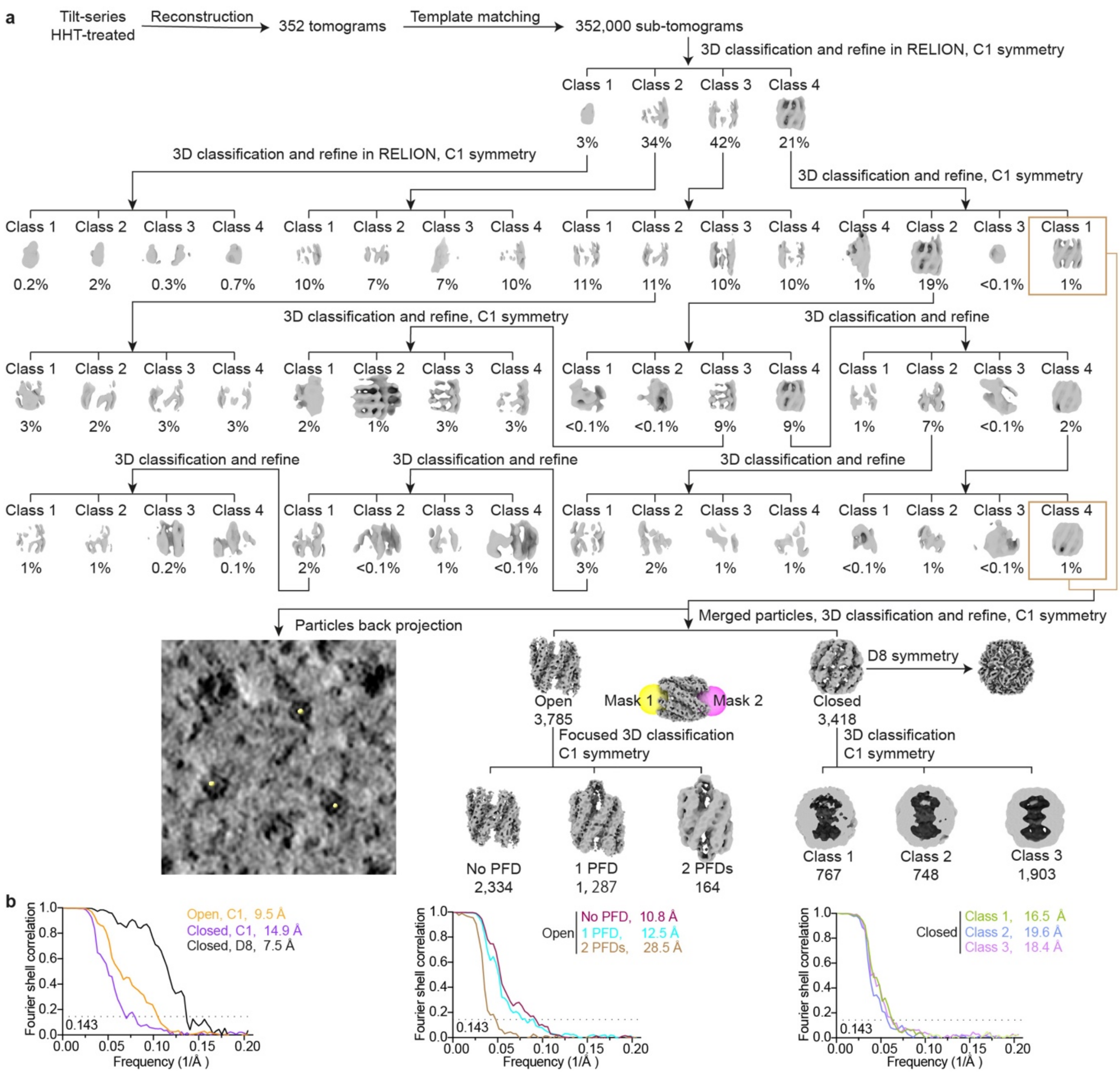

Supplementary Fig. 1: Data processing of TRiC in HHT-treated cells.

a, The image-processing approach of TRiC in the HHT-treated dataset was the same as the untreated dataset (Extended Data Fig. 2a and Methods). ~2% TRiC particles were highlighted in brown square and all percentages in the workflow were shown as the percent of 352,000 initial particles for clarity. **b,** FSC curves of corresponding TRiC states and the resolution were displayed (FSC = 0.143).

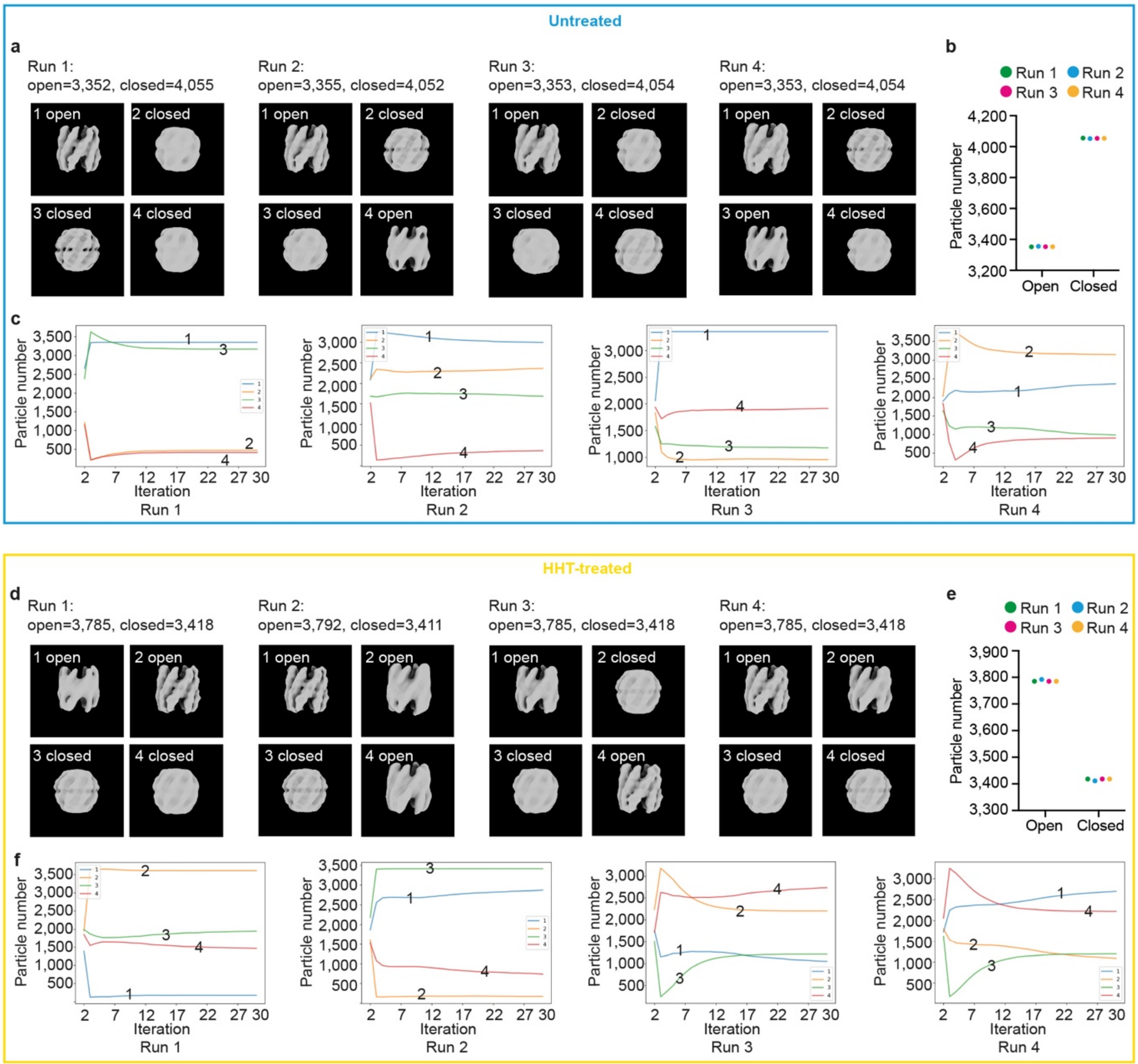

Supplementary Fig. 2: Validation of open and closed TRiC classification in two datasets. **a**, Output of four runs to classify open and closed TRiC from 7,407 TRiC particles in the untreated dataset (classes = 4, T = 0.5, iterations = 30, C1 symmetry, without mask). **b**, In each run in (**a**), the total number of the classified open and closed TRiC was summarized. **c**, The curves show the change in the particle number of each class over 30 iterations in (**a**). **d**, Output of four runs to classify open and closed TRiC from 7,203 TRiC particles in the HHT-treated dataset (classes = 4, T = 0.5, iterations = 30, C1 symmetry, without mask). **e**, In each run in (**d**), the total number of the classified open and closed TRiC was summarized. **f**, The curves show the change in the particle number of each class over 30 iterations in (**d**).

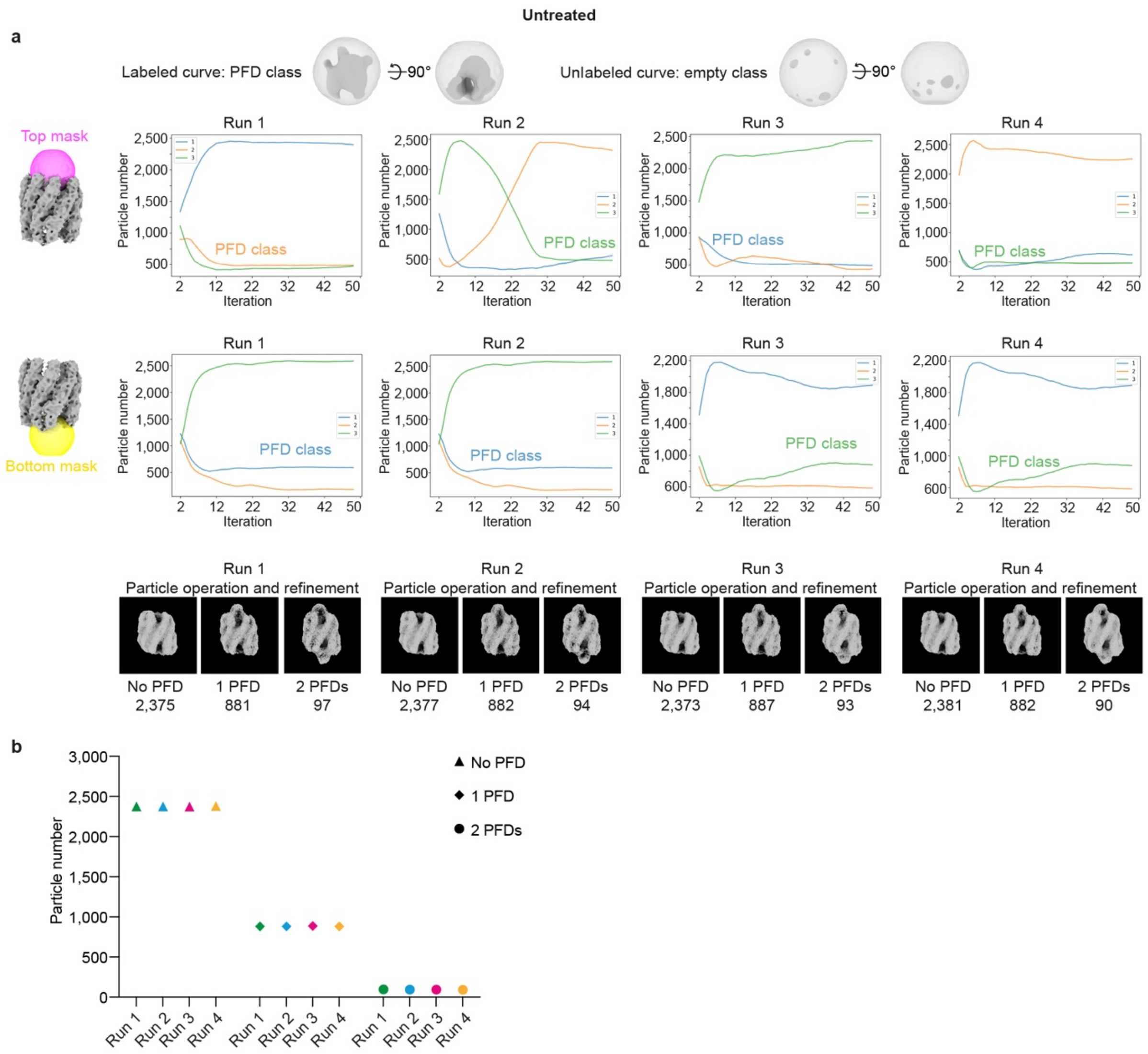

Supplementary Fig. 3: Validation of classification of PFD-bound TRiC particles in untreated cells.

a, Output of four runs to classify TRiC without PFD, with 1 PFD and 2 PFDs from 3,353 open TRiC particles with a sphere mask focused on the top PFD region or bottom PFD region (classes = 3, T = 3, iterations = 50, C1 symmetry)**.** The curves show the change in the particle number of each class over 50 iterations. PFD-bound class was labeled and empty classes were not labeled on the curve for virilization. The TRiC classes without PFD, with 1 PFD and 2 PFDs were sorted by finding shared and unique particles from focused classifications with the top and bottom masks (Methods). **b**, In each run in (**a**), the total number of TRiC without PFD and with PFD was summarized.

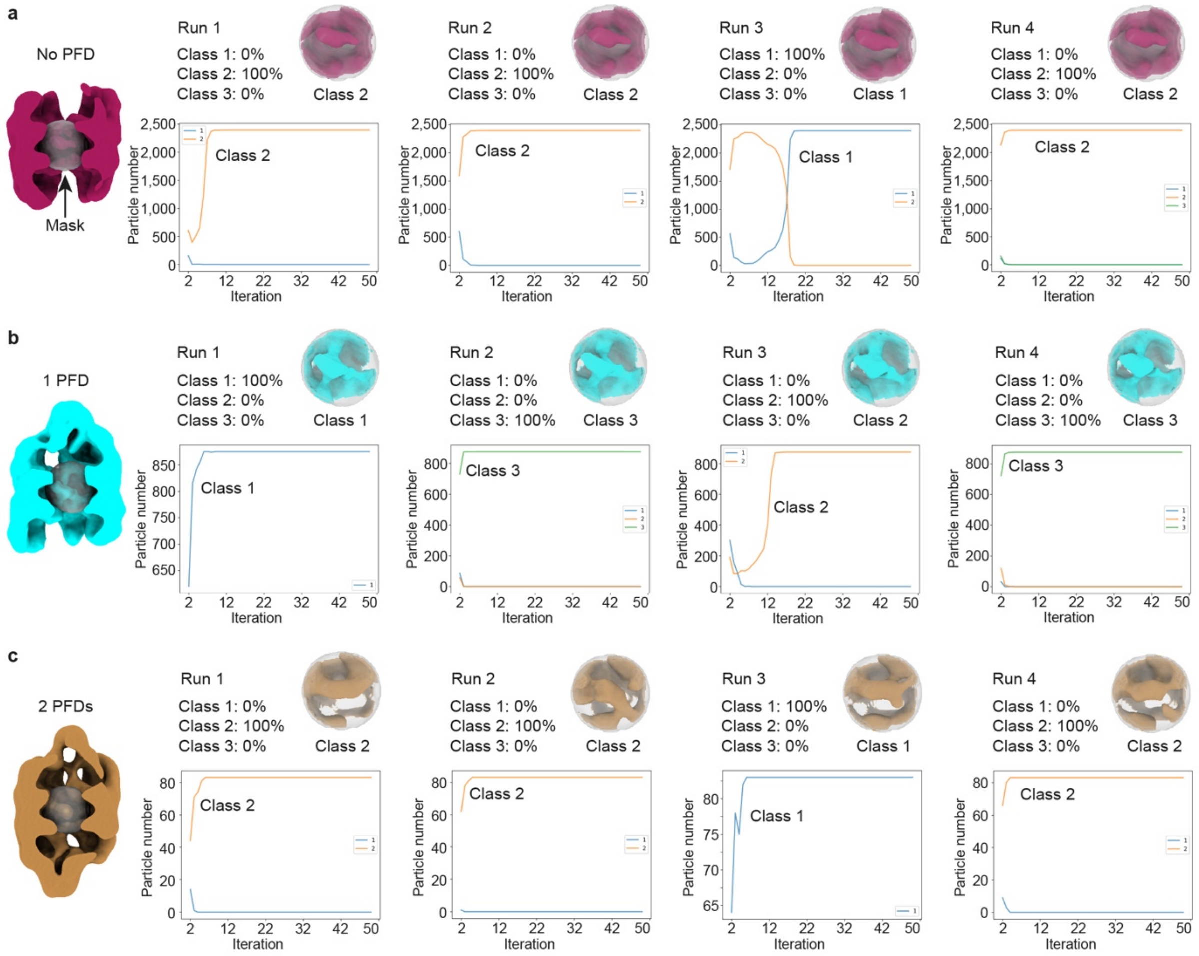

Supplementary Fig. 4: Classification of densities in the open TRiC chamber.

a-c, Multiple runs of focused classification with a mask covering the densities in the TRiC chamber. The curves show the change in the particle number of each class over 50 iterations. **a**, open TRiC without PFD bound. **b**, open TRiC with 1 PFD bound. **c**, open TRiC with 2 PFDs bound. Run 1 (T = 3, 3 classes, 50 iterations), Run 2 (T = 1, 3 classes, 50 iterations), Run 3 (T = 5, 3 classes, 50 iterations) and Run 4 (T = 7, 3 classes, 50 iterations) in (**a-c**).

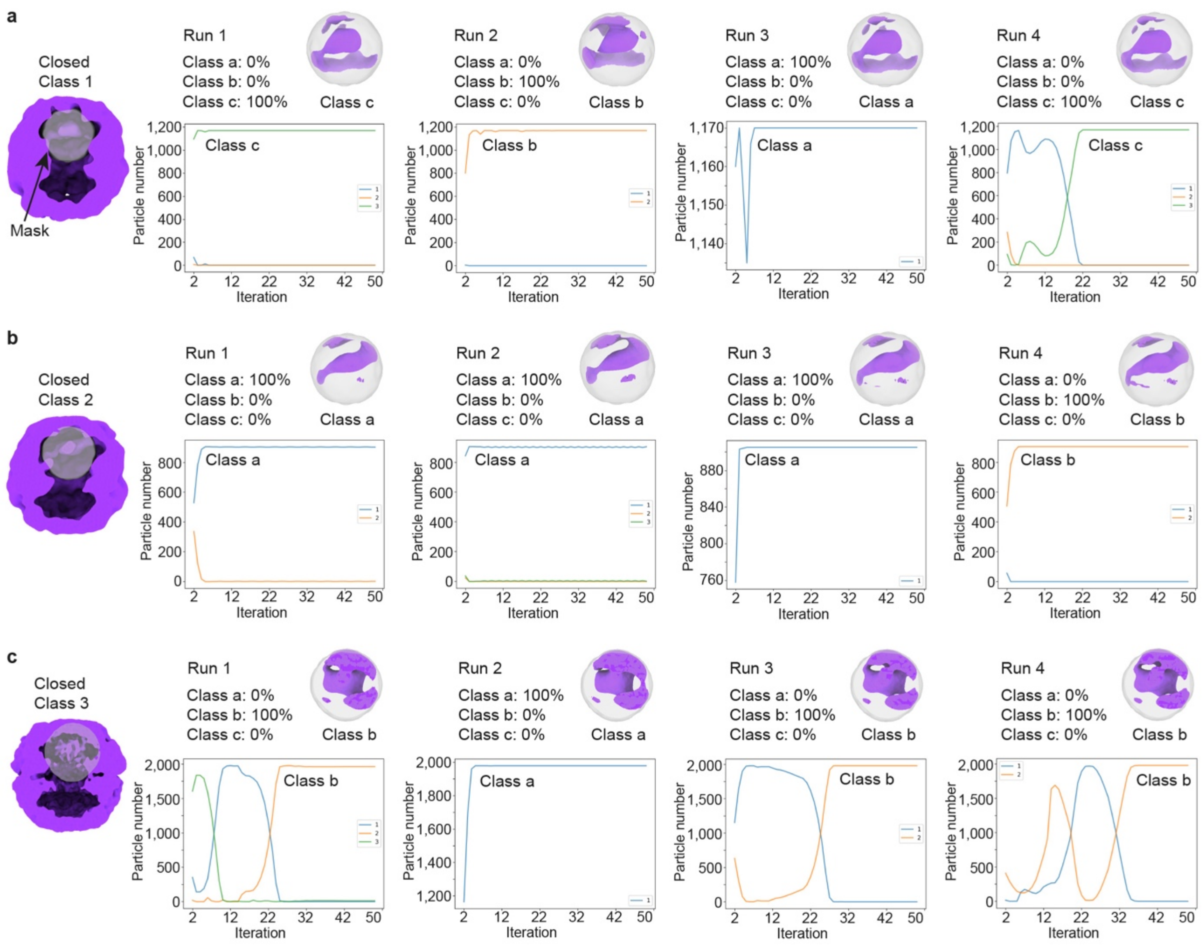

Supplementary Fig. 5: Classification of densities in the closed TRiC chamber.

a-c, Multiple runs of focused classification with a mask covering the densities in the TRiC chamber. The curves show the change in the particle number of each class over 50 iterations. **a**, Closed TRiC-class 1. **b**, Closed TRiC-class 2. **c**, Closed TRiC-class 3. Run 1 (T = 3, 3 classes, 50 iterations), Run 2 (T = 1, 3 classes, 50 iterations), Run 3 (T = 5, 3 classes, 50 iterations) and Run 4 (T = 7, 3 classes, 50 iterations) in (**a-c**).

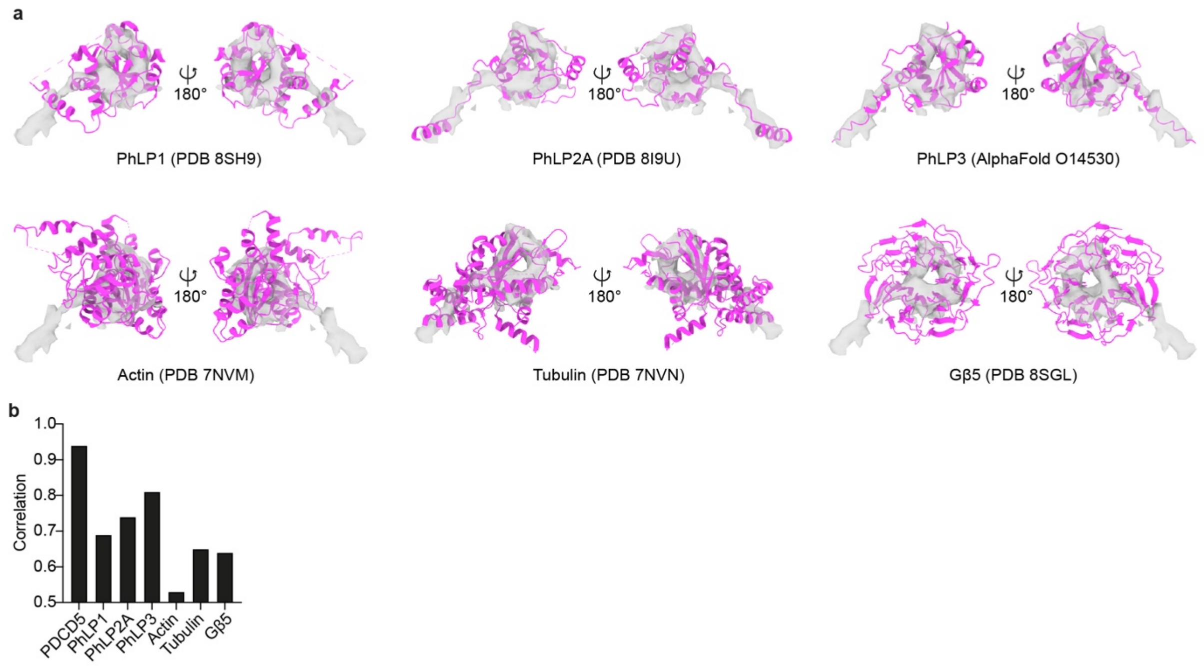

Supplementary Fig. 6: The additional density from open TRiC fitted with TRiC binders. **A**, Atomic model of PhLP1, PhLP2A, PhLP3, actin, tubulin and Gβ5 were fitted into the additional density segmented from the open TRiC map. PhLP1 (79-277 a.a. out of full length 301 a.a.), PhLP2A (91-232 a.a. out of full length 239 a.a.), PhLP3 (68-209 a.a. out of full length 226 a.a.) were used to achieve the best fitting for calculating the correlation. **B**, Calculation of correlation of the fitting shown in (**a**) in ChimeraX. The map simulated from atoms of the PDB model is at the resolution of 8 Å, which is similar to our map resolution. The fitting of PDCD5 was shown in Extended Data Fig. 5e.

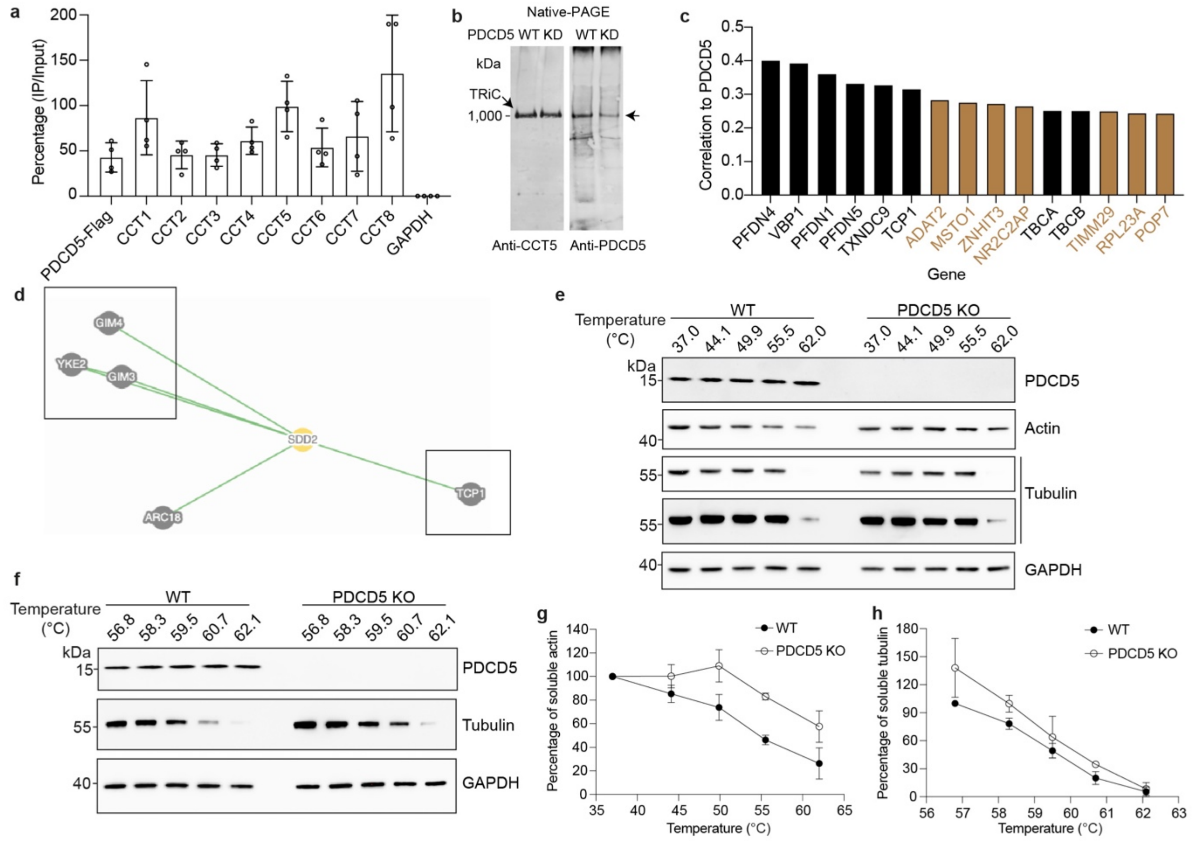

Supplementary Fig. 7: Functional association of PDCD5 with the TRiC and its substrates. **a**, Efficiency of the co-IP in (Fig. 2c). The percentage was calculated by IP/Input and normalized by the amount of loaded sample (Input 1% and IP 5%) for western. The percentage's variability may depend on how many unassembled CCT subunits and assembled TRiC complexes in the cell lysis (Input). The data represent the mean ± SD of four independent experiments (Methods). **b**, Native gel analysis of cell lysis from HEK cell. WT, wild-type. KD, knockdown PDCD5. The arrowhead shows PDCD5 comigrates with TRiC. **c,** Top 15 genes associated with PDCD5 based on the analysis in DepMap (https://depmap.org). Genes involved in protein folding were colored in black. PFDN4 (prefoldin subunit 4), VBP1 (prefoldin subunit 3), PFDN1 (prefoldin subunit 1), PFDN5 (prefoldin subunit 5), TXNDC9 (thioredoxin domain-containing protein 9), TCP1 (CCT1), TBCA (Tubulin-specific chaperone A), TBCB (Tubulin-folding cofactor B). The y-axis represents the Pearson correlation. **d,** Genetic interaction network of SDD2 generated in Saccharomyces Genome Database

(https://www.yeastgenome.org/). The interactions were filtered by three experiments. Genes directly relevant to folding were marked with black squares. ARC18 is involved in the regulation of actin polymerization. **e, f**, Western blot images of the soluble fraction of actin and tubulin at different temperatures in wild-type and PDCD5 knockout HEK 293T cells. Two western blotting images of tubulin showing short and long exposure times in (**e**). **g**, Quantification of soluble actin in (**e**) at corresponding temperatures. The density values of actin were normalized by GAPDH. The data represent the mean ± SD of two independent experiments (Methods). **h**, Quantification of soluble tubulin in (**f**). The density values of tubulin were normalized by GAPDH. The data represent the mean ± SD of two independent experiments (Methods).

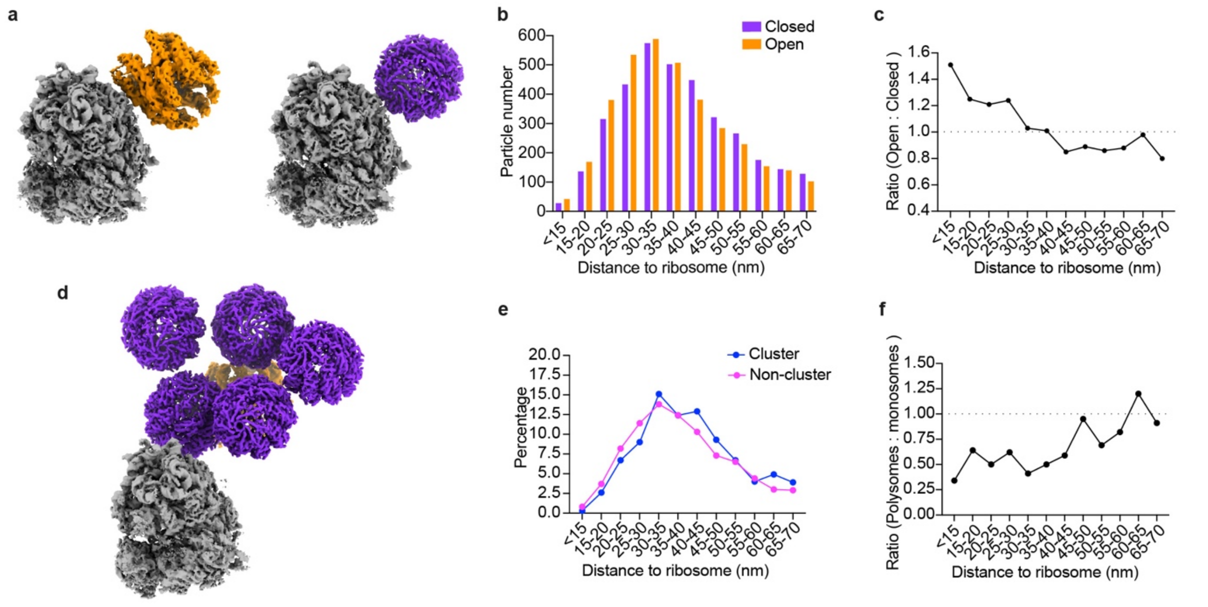

Supplementary Fig. 8: Spatial analysis of TRiC and ribosome in human cells.

a, Snapshots of the open and closed TRiC close to the ribosome exit tunnel. **b**, Distribution of the number of open and closed TRiC according to the distance to neighboring ribosomes (TRiC center to the ribosome exit tunnel). The number of open TRiC was normalized by the number of closed TRiC for comparison. Normalized $Open_{distance} = Open_{distance} * (closed\text{-}all/open\text{-}all) = Open_{distance} *(4{,}054/3{,}353)$. **c**, The ratio of open and closed TRiC with neighboring ribosomes in (**b**). **d**, A snapshot of a TRiC cluster close to the ribosome exit tunnel. **e**, Percentage of non-clustered TRiC and clustered TRiC with ribosome neighbors in various distances (TRiC center to the ribosome exit tunnel). **f**, Normalized ratio of polysomes and monosomes with neighboring TRiC clusters at different distances. The distance was measured between the TRiC center and the ribosome exit tunnel.

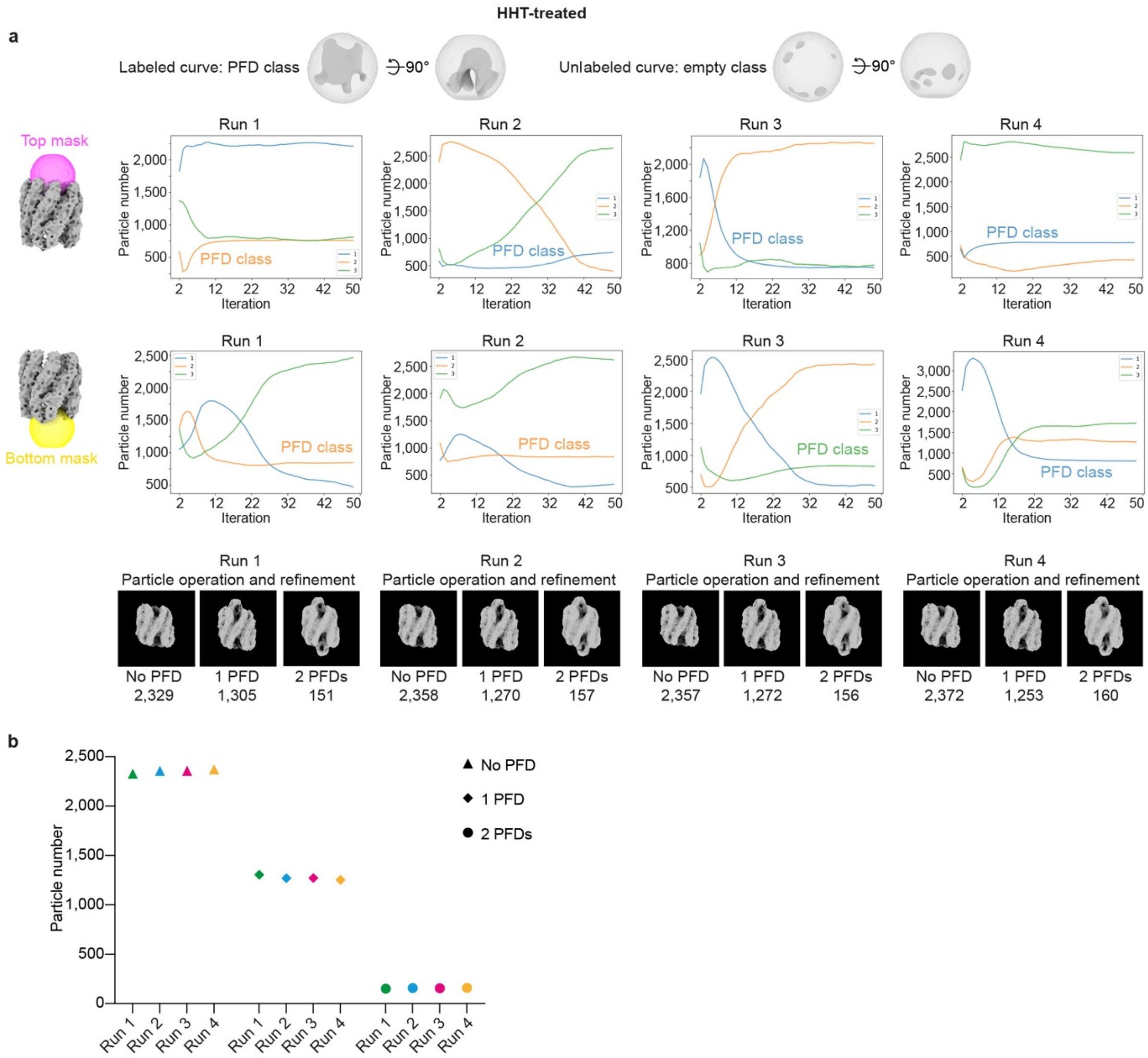

Supplementary Fig. 9: Validation of classification of PFD-bound TRiC particles in HHT-treated cells.

a, Output of four runs to classify TRiC without PFD, with 1 PFD and 2 PFDs from 3,785 open TRiC particles with a sphere mask focused on the top PFD or bottom PFD region (classes = 3, T = 3, iterations = 50, C1 symmetry)**.** The curves show the change in the particle number of each class over 50 iterations. PFD-bound class was labeled and empty classes were not labeled on the curve. The TRiC classes without PFD, with 1 PFD and 2 PFDs were sorted by finding shared and unique particles from focused classifications with the top and bottom masks (Methods). **b**, In each run in (**a**), the total number of TRiC without PFD and with PFD was summarized.

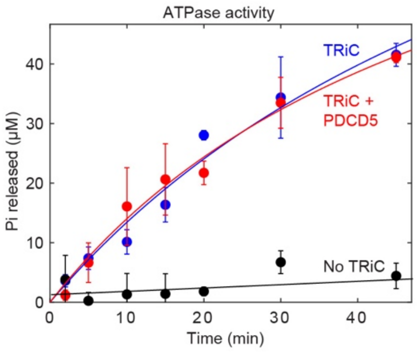

Supplementary Fig. 10: PDCD5 does not affect the ATPase activity of TRiC

a, ATPase activity of 0.3 μM TRiC in the absence and presence of 3 μM PDCD5 by quinaldine red assay measuring Pi release over time. Data are presented as mean values ± SEM (n = 2 biologically independent experiments).

Supplementary Video 1-9 (provided separately)
