# Supplementary material for: In situ analysis reveals the TRiC duty cycle and PDCD5 as an open-state cofactor
Source: Nature. 2024 Dec 11;637(8047):983–90. doi: 10.1038/s41586-024-08321-z (PMC11754096; doi:10.1038/s41586-024-08321-z)
Supplement: Supplementary file 2 — Reporting Summary [file 41586_2024_8321_MOESM2_ESM.pdf]

nature portfolio

Corresponding author(s): Martin Beck
Judith Frydman

Last updated by author(s): Oct 5, 2024

Reporting Summary

Nature Portfolio wishes to improve the reproducibility of the work that we publish. This form provides structure for consistency and transparency in reporting. For further information on Nature Portfolio policies, see our Editorial Policies and the Editorial Policy Checklist.

Statistics

For all statistical analyses, confirm that the following items are present in the figure legend, table legend, main text, or Methods section.

n/a	Confirmed	
☐	☒	The exact sample size (*n*) for each experimental group/condition, given as a discrete number and unit of measurement
☐	☒	A statement on whether measurements were taken from distinct samples or whether the same sample was measured repeatedly
☐	☒	The statistical test(s) used AND whether they are one- or two-sided<br>*Only common tests should be described solely by name; describe more complex techniques in the Methods section.*
☒	☐	A description of all covariates tested
☒	☐	A description of any assumptions or corrections, such as tests of normality and adjustment for multiple comparisons
☐	☒	A full description of the statistical parameters including central tendency (e.g. means) or other basic estimates (e.g. regression coefficient) AND variation (e.g. standard deviation) or associated estimates of uncertainty (e.g. confidence intervals)
☐	☒	For null hypothesis testing, the test statistic (e.g. *F*, *t*, *r*) with confidence intervals, effect sizes, degrees of freedom and *P* value noted<br>*Give P values as exact values whenever suitable.*
☒	☐	For Bayesian analysis, information on the choice of priors and Markov chain Monte Carlo settings
☒	☐	For hierarchical and complex designs, identification of the appropriate level for tests and full reporting of outcomes
☒	☐	Estimates of effect sizes (e.g. Cohen's *d*, Pearson's *r*), indicating how they were calculated

Our web collection on statistics for biologists contains articles on many of the points above.

Software and code

Policy information about availability of computer code

Data collection	Tilt series were collected using SerialEM version 4.0.1.
Data analysis	For subtomogram averaging and classification, we used IMOD 4.11.0, Warp 1.0.9 and RELION 3.1.<br>For structure and tomogram visualization, we used UCSF ChimeraX-1.6.1 and napari (version 0.4.16).<br>We used cryoCAT package for the spatial analysis of TRiC (https://github.com/turonova/cryoCAT).<br>Protein structure prediction was performed with AlphaFold-Multimer 2.2.0.<br>Data plotting and statistical analysis were performed using GraphPad Prism (version 10, GraphPad Software).<br>Protein sequence alignment was performed with Clustal Omega (https://www.ebi.ac.uk/jdispatcher/msa/clustalo) or ClustalO in Jalview6 (Version: 2.11.4.0).

For manuscripts utilizing custom algorithms or software that are central to the research but not yet described in published literature, software must be made available to editors and reviewers. We strongly encourage code deposition in a community repository (e.g. GitHub). See the Nature Portfolio guidelines for submitting code & software for further information.

Data

Policy information about availability of data

All manuscripts must include a data availability statement. This statement should provide the following information, where applicable:

- Accession codes, unique identifiers, or web links for publicly available datasets
- A description of any restrictions on data availability
- For clinical datasets or third party data, please ensure that the statement adheres to our policy

Cryo-ET maps have been deposited to the Electron Microscopy Data Bank (EMDB) under accession numbers EMD-18921 (Open TRiC in untreated and HHT-treated cells), EMD-18913 (Closed TRiC in untreated and HHT-treated cells, C1 symmetry), EMD-18914 (Closed TRiC in untreated and HHT-treated cells, D8 symmetry), EMD-18922 (Open TRiC in untreated cells), EMD-18923 (Open TRiC without PFD in untreated cells), EMD-18924 (Open TRiC with one PFD in untreated cells), EMD-18925 (Open TRiC with two PFDs in untreated cells), EMD-18926 (Closed TRiC in untreated cells, C1 symmetry), EMD-18927 (Closed TRiC in untreated cells, D8 symmetry), EMD-18928 (Closed TRiC-class 1 in untreated cells), EMD-18929 (Closed TRiC-class 2 in untreated cells), EMD-18930 (Closed TRiC-class 3 in untreated cells), EMD-18931 (Open TRiC in HHT-treated cells), EMD-18932 (Open TRiC without PFD in HHT-treated cells), EMD-18933 (Open TRiC with one PFD in HHT-treated cells), EMD-18934 (Open TRiC with two PFDs in HHT-treated cells), EMD-18936 (Closed TRiC in HHT-treated cells, C1 symmetry), EMD-18937 (Closed TRiC in HHT-treated cells, D8 symmetry), EMD-18938 (Closed TRiC-class 1 in HHT-treated cells), EMD-18939 (Closed TRiC-class 2 in HHT-treated cells) and EMD-18940 (Closed TRiC-class 3 in HHT-treated cells). Maps and atomic models used from previous studies were downloaded from the EMDB (EMD-12606, EMD-12607 and EMD-40461) and the PDB (2K6B, 7X3J, 7NVN, 7NVO, 7NVL, 7NVM, 8F8P and 7WU7). The model of PDCD5 was from the AlphaFold Protein Structure Database (AF-O14737-F1). Saccharomyces Genome Database is available at https://www.yeastgenome.org/. Protein sequences were from UniProt: CCT1-8 (UniProt: P17987, P78371, P49368, P50991, P48643, P40227, Q99832 and P50990), PDCD5 (UniProt: M.maripaludis_A9A8D7, S.pombe_O13929, C.elegans_Q93408, Mouse_P56812, Bovine_Q2HJH9, Human_O14737) and CCT1 (UniProt: H.volcanii_O30561, S.pombe_O94501, C.elegans_P41988, Mouse_P11983, Bovine_Q32L40, Human_P17987). Source data (raw gels and blots) to Fig.2c, Extended Data Figs. 6f,h,i and 7g,i,k , and Supplementary Fig 7b,e,f are provided with this paper.

Research involving human participants, their data, or biological material

Policy information about studies with human participants or human data. See also policy information about sex, gender (identity/presentation), and sexual orientation and race, ethnicity and racism.

Reporting on sex and gender	N/A
Reporting on race, ethnicity, or other socially relevant groupings	N/A
Population characteristics	N/A
Recruitment	N/A
Ethics oversight	N/A

Note that full information on the approval of the study protocol must also be provided in the manuscript.

Field-specific reporting

Please select the one below that is the best fit for your research. If you are not sure, read the appropriate sections before making your selection.

☒ Life sciences ☐ Behavioural & social sciences ☐ Ecological, evolutionary & environmental sciences

For a reference copy of the document with all sections, see nature.com/documents/nr-reporting-summary-flat.pdf

Life sciences study design

All studies must disclose on these points even when the disclosure is negative.

Sample size

The cryo-ET sample sizes were not predetermined and were limited by the availability of microscopy time. These cryo-ET data were sufficient to support our conclusions based on the resolution of the cryo-ET maps. Additionally, the datasets used in this study represent one of the largest cryo-ET datasets of human cells at this pixel size. The number of images collected is indicated in Extended Data Fig. 2 and Supplementary Fig. 1. Sample sizes for the cryo-ET map obtained in this study: Structure of the open TRiC in untreated and HHT-treated cells was determined from 7138 particles. Structure of the closed TRiC- C1 symmetry in untreated and HHT-treated cells was determined from 7472 particles. Structure of the closed TRiC- D8 symmetry in untreated and HHT-treated cells was determined from 7472 particles. Structure of the open TRiC in untreated cells was determined from 3353 particles. Structure of the open TRiC without PFD in untreated cells was determined from 2395 particles. Structure of the open TRiC with one PFD in untreated cells was determined from 875 particles. Structure of the open TRiC with two PFDs in untreated cells was determined from 83 particles. Structure of the closed TRiC-C1 symmetry in untreated cells was determined from 4054 particles. Structure of the closed TRiC-D8 symmetry in untreated cells was determined from 4054 particles. Structure of the closed TRiC-class 1 in untreated cells was determined from 1170 particles. Structure of the closed TRiC-class 2 in untreated cells was determined from 905 particles. Structure of the closed TRiC-class 3 in untreated cells was determined from 1979 particles. Structure of the open TRiC in HHT-treated cells was determined from 3785 particles. Structure of the open TRiC without PFD in HHT-treated cells was determined from 2334 particles. Structure of the open TRiC with one PFD in HHT-treated cells was determined from 1287 particles. Structure

of the open TRiC with two PFDs in HHT-treated cells was determined from 164 particles. Structure of the closed TRiC-C1 symmetry in HHT-treated cells was determined from 3418 particles. Structure of the closed TRiC-D8 symmetry in HHT-treated cells was determined from 3418 particles. Structure of the closed TRiC-class 1 in HHT-treated cells was determined from 767 particles. Structure of the closed TRiC-class 2 in HHT-treated cells was determined from 748 particles. Structure of the closed TRiC-class 3 in HHT-treated cells was determined from 1903 particles. Biochemical sample sizes were not predetermined but were determined after completing three independent replicates and evaluating statistical significance to ensure reproducibility.

Data exclusions

In cryo-ET image processing, the elimination of particles erroneously identified as valid peaks is achieved through 3D classification procedures. This step is a standard practice in cryo-EM image processing.

Replication

Cells grow on the EM grids in four independent cell culture dishes before plunge freezing for the untreated dataset. Cells grow on the EM grids in four independent cell culture dishes before plunge freezing for the HHT-treated dataset. The pipeline used in this study is reproducible.

Randomization

Randomization was not required because all data were used for the analysis in this study.

Blinding

This study focuses on structural and spatial analysis of a cellular machine, which does not involve any experiments related to blinding.

Reporting for specific materials, systems and methods

We require information from authors about some types of materials, experimental systems and methods used in many studies. Here, indicate whether each material, system or method listed is relevant to your study. If you are not sure if a list item applies to your research, read the appropriate section before selecting a response.

Materials & experimental systems

n/a	Involved in the study	
☐	☒	Antibodies
☐	☒	Eukaryotic cell lines
☒	☐	Palaeontology and archaeology
☒	☐	Animals and other organisms
☒	☐	Clinical data
☒	☐	Dual use research of concern
☒	☐	Plants

Methods

n/a	Involved in the study	
☒	☐	ChIP-seq
☒	☐	Flow cytometry
☒	☐	MRI-based neuroimaging

Antibodies

Antibodies used

mouse anti-FLAG M2 (Sigma-Aldrich, F1804, 1:2,000), rabbit anti-PDCD5 (abcam, ab126213, 1:1,000), rabbit anti-CCT1 (abcam, ab240903, 1:10,000), rabbit anti-CCT2 (abcam, ab92746, 1:10,000), rabbit anti-CCT3 (proteintech, 10571-1-AP, 1:30,000), rabbit anti-CCT4 (proteintech, 21524-1-AP, 1:5,000), rabbit anti-CCT5 (proteintech, 11603-1-AP, 1:3,000), rabbit anti-CCT6 (proteintech, 19793-1-AP, 1:1,000), rabbit anti-CCT7 (abcam, ab240566, 1:30,000), rabbit anti-CCT8 (proteintech, 12263-1-AP, 1:2,000), rabbit anti-GAPDH (proteintech, 10494-1-AP, 1:15,000), mouse anti-actin (Invitrogen, AM4302, 1:3,000), mouse anti-tubulin (Sigma, T5168, 1:3,000), anti-rabbit IgG (Cell signaling, 7074, 1:10,000), anti-mouse IgG + IgM (Jackson ImmunoResearch, 115-035-044, 1:10,000), rabbit anti-PDCD5 (proteintech, 12456-1-AP, 1:1,000), mouse anti-CCT8 (Santa Cruz Biotechnology, sc-377261, 1:250), rabbit anti-CCT5 (abcam, ab129016,1:10,000)

Validation

mouse anti-FLAG M2 (Sigma-Aldrich, F1804), https://www.sigmaaldrich.com/DE/en/product/sigma/f1804?=kr&srsltid=AfmBOoqeCslWG5-kRE-f0WnSaQYNp5wxVrHDkOJMfNEkgca97KGtzCR8
rabbit anti-PDCD5 (abcam, ab126213), https://www.abcam.com/en-us/products/primary-antibodies/pdcd5-antibody-ab126213
rabbit anti-CCT1 (abcam, ab240903), https://www.abcam.com/en-us/products/primary-antibodies/tcp1-alpha-ccta-antibody-91a-ab240903
rabbit anti-CCT2 (abcam, ab92746), https://www.abcam.com/en-us/products/primary-antibodies/cct2-antibody-epr4084-ab92746
rabbit anti-CCT3 (proteintech, 10571-1-AP), https://www.ptglab.com/products/CCT3-Antibody-10571-1-AP.htm
rabbit anti-CCT4 (proteintech, 21524-1-AP), https://www.ptglab.com/products/CCT4-Antibody-21524-1-AP.htm
rabbit anti-CCT5 (proteintech, 11603-1-AP), https://www.ptglab.com/products/CCT5-Antibody-11603-1-AP.htm
rabbit anti-CCT6 (proteintech, 19793-1-AP), https://www.ptglab.com/products/CCT6A-Specific-Antibody-19793-1-AP.htm
rabbit anti-CCT7 (abcam, ab240566), https://www.abcam.com/en-us/products/primary-antibodies/tcp1-eta-antibody-ab240566
rabbit anti-CCT8 (proteintech, 12263-1-AP), https://www.ptglab.com/products/CCT8-Antibody-12263-1-AP.htm
rabbit anti-GAPDH (proteintech, 10494-1-AP)), https://www.ptglab.com/products/GAPDH-Antibody-10494-1-AP.htm
mouse anti-actin (Invitrogen, AM4302), https://www.thermofisher.com/antibody/product/beta-Actin-Antibody-clone-AC-15-Monoclonal/AM4302
mouse anti-tubulin (Sigma, T5168), https://www.sigmaaldrich.com/DE/en/product/sigma/t5168
rabbit anti-PDCD5 (proteintech, 12456-1-AP), https://www.ptglab.com/products/PDCD5-Antibody-12456-1-AP.htm
mouse anti-CCT8 (Santa Cruz Biotechnology, sc-377261), https://www.scbt.com/p/tcp-1-theta-antibody-e-7?srsltid=AfmBOoo6lhdT8i2Hq_gFUwDuwfwbaFbPgRgnBU-9JAwbmJtuUuFCX7yL
rabbit anti-CCT5 (abcam, ab129016), https://www.abcam.com/en-us/products/primary-antibodies/tcp1-epsilon-cct5-antibody-epr7562-ab129016

Eukaryotic cell lines

Policy information about cell lines and Sex and Gender in Research

Cell line source(s)	HEK Flp-In T-Rex 293 (Invitrogen), HEK293F (Thermo Fisher), Wide-type HEK293T (abcam, ab255449), PDCD5 knockout HEK 293T cells (abcam, ab266229)
Authentication	No additional authentication was conducted for commercially available cell lines.
Mycoplasma contamination	The cells were tested negative for mycoplasma contamination.
Commonly misidentified lines (See ICLAC register)	No commonly misidentified cell lines were used in the study.

Plants

Seed stocks	N/A
Novel plant genotypes	N/A
Authentication	N/A
